# Correction to: COVID-19 vaccine preferences among university students in Hong Kong: a discrete choice experiment

**DOI:** 10.1186/s13104-021-05889-x

**Published:** 2021-12-31

**Authors:** Xue Li, Man Yui Chong, Ching Yui Chan, Vindy Wing Sum Chan, Xinning Tong

**Affiliations:** 1 Department of Medicine, Li Ka Shing Faculty of Medicine, The University of Hong Kong, Hong Kong, People’s Republic of China; 2 Centre for Safe Medication Practice and Research, Department of Pharmacology and Pharmacy, Li Ka Shing Faculty of Medicine, The University of Hong Kong, Hong Kong, People’s Republic of China; 3 Li Ka Shing, Faculty of Medicine, University of Hong Kong, Hong Kong, People’s Republic of China

## Correction to: BMC Research Notes (2021) 14:421 https://doi.org/10.1186/s13104-021-05841-z

Following publication of the original article [1], the authors identified an error in the author name of Vindy Wing Sum Chan.

The incorrect author name is: Vindy Wing Sun Chan.

The correct author name is: Vindy Wing Sum Chan.

The author group has been updated above and the original article [1] has been corrected.
